# Endovascular thrombectomy versus intravenous tissue plasminogen activator for vertebrobasilar stroke treatment: insights from the national inpatient sample

**DOI:** 10.3389/fneur.2025.1417188

**Published:** 2025-04-24

**Authors:** Ram Saha, Gaurav Nepal, Dhanshree Solanki, Ahmed Shaheen, Mohammed Maan Al-Salihi, Shamser Singh Dalal, Anil Roy

**Affiliations:** ^1^Department of Neurology Virginia, Commonwealth University, Richmond, VA, United States; ^2^Department of Neurology, University Hospitals Cleveland Medical Centre and Case Western Reserve University, Cleveland, OH, United States; ^3^Research Update Organization, Houston, TX, United States; ^4^Alexandria Faculty of Medicine, Alexandria, Egypt; ^5^Zeenat Qureshi Stroke Institute, University of Missouri, Columbia, MO, United States; ^6^Department of Radiology, University of Virginia, Charlottesville, VA, United States; ^7^Department of Neurosurgery, Virginia Commonwealth University, Richmond, VA, United States

**Keywords:** endovascular thrombectomy, intravenous tissue plasminogen activator, IV-tPA, vertebrobasilar artery, vertebrobasilar stroke

## Abstract

**Introduction:**

Approximately 20% of patients, who present with acute ischemic stroke are diagnosed with acute vertebrobasilar artery occlusion (VBAO), which is caused by an embolus or ruptured atherosclerotic plaque leading to the formation of an acute thrombus. The mortality rate of VBAO is extremely high without treatment, ranging from 80 to 95%, underscoring the urgent need for effective and timely treatment strategies. In this study, we examined the trends of hospitalizations for Endovascular Thrombectomy (EVT) or intravenous tissue plasminogen activator (IV-tPA) as interventions for VBAO, their outcomes, associated complications, and predictors of mortality in patients undergoing these procedures.

**Methods:**

We utilized the National Inpatient Sample (NIS) database to extract data from the years 2016 to 2018, using ICD-10 diagnosis and procedure codes specific to occlusion or thrombosis of the vertebral artery or basilar artery, IV-tPA, and EVT.

**Results:**

Between 2016 and 2018, a total of 37,310 patients were admitted with VBAO. Among these, tPA was administered in 2,530 admissions (6.8%), while EVT was performed in 2,330 admissions (6.2%). IV-tPA was more frequently used in the age groups of 65–84 years and, ≥85 years, whereas EVT was more commonly used in the age groups of 18–44 years and 45–64 years. There was no significant difference in usage between men and women. In large hospitals, EVT was more commonly used than IV-tPA (8.1% vs. 7%, *p* < 0.0001), while in small hospitals, IV-tPA usage was significantly higher (3.8% vs. 2%, *p* < 0.0001). The all-cause mortality rate was significantly higher in EVT admissions compared to IV-tPA admissions (16.8% vs. 8.1%, *p* < 0.0001). However, there was no significant difference in the mean length of stay (LOS) between the two modalities.

**Conclusion:**

A trend of higher rates of EVT was observed in the younger age group (18–64 years) compared to the older age group, but no significant difference was noted based on sex. The all-cause mortality rate was found to be higher in the EVT group compared to the IV-tPA group. However, there was no significant difference in the length of hospital stay between the two groups.

## Introduction

Diagnosing posterior circulation strokes and TIAs can be trickier due to the diverse and subtle symptoms they present (1). The debate between endovascular therapy (EVT) and medical therapy for acute ischemic stroke (AIS) caused by vertebrobasilar artery occlusion (VBAO) is an ongoing challenge. The consequences of VBAO can be severe, and the urgency of early reperfusion is crucial to minimize the associated mortality and morbidity (2, 3). Recognizing and addressing the need for optimal management in posterior circulation strokes is vital for comprehensive stroke care (1).

Prior studies have explored treatment modalities for VBAO, highlighting their potential impact on morbidity and mortality. The BASICS trial demonstrated that EVT could offer a marginal survival advantage in patients with basilar artery occlusion when performed within a specific time window, though it did not show a significant difference in functional outcomes compared to standard medical therapy (4). Similarly, the BEST trial revealed the feasibility of EVT in achieving recanalization; however, complications such as procedural-related hemorrhages and delayed neurological recovery remain concerns (5). These findings emphasize the need for further exploration of EVT and intravenous tissue plasminogen activator (IV-tPA) in real-world hospital settings. This study aims to address these gaps by analyzing trends and outcomes associated with EVT and IV-tPA in a large inpatient population.

## Methods

### Data source

Our research utilized a retrospective observational design, analyzing data from the National Inpatient Sample (NIS) database files spanning from 2016 to 2018. The NIS files are published annually by the Agency for Healthcare Research and Quality (AHRQ) as part of the Healthcare Cost and Utilization Project (HCUP) (6, 7). The NIS is the largest all-payer inpatient database in the United States, publicly available, and represents approximately 20% stratified sample of discharges from community hospitals nationwide (7, 8). HCUP provides discharge weights for each record, enabling national estimates to be derived (9). Strict measures are in place to ensure patient information confidentiality in this restricted dataset. In accordance with Health Insurance Portability and Accountability Act (HIPAA) guidelines, Institutional Review Board (IRB) approval was not required for our study, as the use of this dataset for research purposes is exempt from IRB review (10).

### Study population and description of date elements

**Figure 1:**
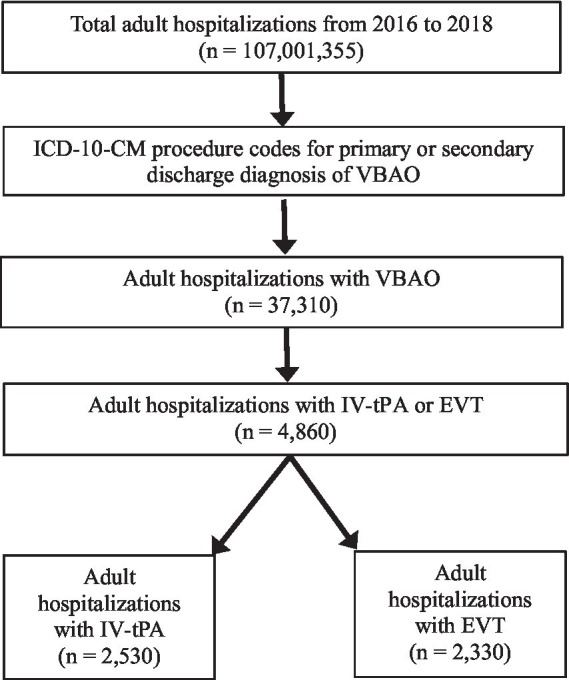
Sequential derivation of the study population.

We identified cases of occlusion or thrombosis of the vertebral artery or basilar artery, IV-tPA administration, and EVT procedures using specific International Classification of Diseases-Tenth Revision-Clinical Modification (ICD-10-CM) diagnosis and procedure codes. We then calculated rates of complications using specific codes. All pertinent codes are listed in Supplementary file 1. In our analysis, patients who underwent both IV-tPA and subsequent EVT were included in the EVT group for statistical purposes. This classification aligns with current clinical practices, which consider EVT as the definitive treatment in combined therapy scenarios due to its mechanical recanalization capability. The overlap between IV-tPA and EVT was accounted for to ensure accurate representation of treatment outcomes, minimizing potential bias in the comparison between the two modalities. In our analysis, we examined multiple variables including age, gender, race, hospital type, hospital location, and disposition status. We also examined additional variables, such as hospital region, median household income, insurance status, and bed size; their description can be found in Supplementary file 2. Discharge disposition was included as a secondary endpoint to assess patient outcomes post-treatment. Patients’ discharge statuses were categorized into the following groups: discharged to home, discharged to a rehabilitation or skilled nursing facility, transferred to another acute care facility, or died during hospitalization. These categorizations were based on data fields available in the NIS database, as outlined in Supplementary file 2. Such categorizations align with standard practices in using administrative datasets for outcome assessment. In the NIS database, diagnoses are listed in a hierarchical manner, with the principal diagnosis being the primary reason for hospitalization, and secondary diagnoses being those that coexist or develop during hospitalization (11). ICD is a standardized coding system that uses alphanumeric codes arranged in a comprehensive and hierarchical manner to define diseases, injuries, and other health-related conditions (12). Our study excluded patients who were under 18 years of age. Figure 1 depicts the rigorous and methodical approach to our statistical analysis.

### Statistical analysis

Study variables were compared using univariate and multivariate analyses. Chi-squared (χ2) test was used to compare categorical variables, while the Wilcoxon rank-sum test was employed for comparing continuous variables (13, 14). Categorical variables were expressed as frequency or percentage, and continuous variables were presented as mean ± error (SE). We used odds ratios (ORs) with 95% confidence intervals (CIs) to report predictors of mortality, which were identified using multivariate regression analysis. For the multivariate regression analyses, we adjusted for the following covariates to control for potential confounding factors: age, gender, race, median household income quartile, hospital characteristics (type, location, and teaching status), primary insurance payer, and region (Northeast, Midwest, South, and West). These covariates were selected based on their relevance to stroke outcomes and treatment utilization patterns, as described in prior studies. These statistical methods have been commonly used in previous studies based on the NIS dataset (15–18). All reported differences in estimates were considered statistically significant (*p* < 0.05) unless otherwise indicated. We used SAS 9.4 (SAS Institute Inc., Cary, North Carolina, USA) software for data analysis.

### Endpoints

Primary endpoints of the study were rates of complications like cerebral edema, intracerebral hemorrhage (ICH), angioedema, hemoperitoneum, arterial dissection, aneurysm of upper or lower extremities, postprocedural hemorrhage or hematoma of skin and subcutaneous tissue following EVT, and postprocedural hemorrhage of a nervous system organ or structure following EVT. Secondary endpoints were all-cause mortality rate and LOS.

## Results

### Number of hospitalizations

**Figure 2:**
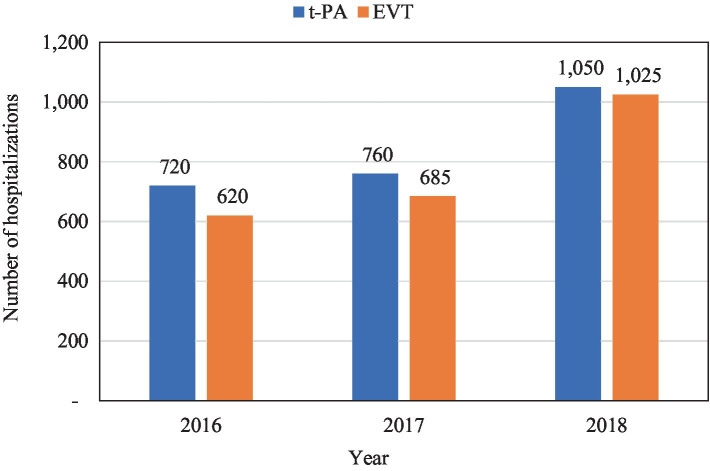
Number of hospitalizations for IV t-PA vs. EVT.

From 2016 to 2018, there were 37,310 patients admitted with VBAO. Out of these, IV-tPA was administered in 2,530 (6.8%) admissions, whereas EVT was done in 2,330 (6.2%) admissions (Table 1; Figure 1). Figure 2 shows the trends in admissions for IV-tPA versus EVT.

**Table 1:** Number of VBAO hospitalizations: no procedure vs. IV-tPA vs. EVT.

	2016	2017	2018	Total	Percentage
No procedure	10,015	11,215	11,220	32,450	87.0
IV t-PA	720	760	1,050	2,530	6.8
EVT	620	685	1,025	2,330	6.2
Total VBAO hospitalizations (*n*)	11,355	12,660	13,295	37,310	

### Baseline characteristics

There was a statistically significant difference in the utilization of IV-tPA and EVT based on age groups (Table 2). IV-tPA was used significantly more frequently in patients older than 65 years, whereas EVT was utilized significantly more often in adult patients under 64 years. These findings reflect established trends in stroke treatment where EVT, requiring specialized expertise, is often preferred in younger patients with fewer comorbidities and greater procedural tolerance. In contrast, IV-tPA is more commonly administered in older patients who may not be eligible for EVT due to medical contraindications or anatomical considerations. There were no statistically significant differences based on gender in the utilization of IV-tPA or EVT. There was no statistically significant difference in the utilization of IV-tPA or EVT for VBAO between White patients and Hispanics patients. However, EVT was used significantly more frequently than IV-tPA in Black patients, with a utilization rate of 6.3% compared to 5.4% (*p* = 0.0001). IV-tPA was utilized more often than EVT irrespective of the hospital bed size. In Midwestern hospitals, EVT was utilized more frequently than IV-tPA, with utilization rates of 6.4 and 5.9%, respectively (*p* = 0.002). On the other hand, in Western hospitals, IV-tPA was used more often than EVT, with utilization rates of 9.5 and 6.9%, respectively (*p* < 0.0001). However, no statistically significant differences were observed in Northeastern and Southern hospitals in terms of IV-tPA and EVT utilization for VBAO. In rural and urban non-teaching hospitals, IV-tPA was utilized more frequently than EVT for VBAO. However, in teaching hospitals, EVT was utilized more often than IV-tPA, with utilization rates of 5.7 and 5.6%, respectively (*p* < 0.0001). The observed variation in treatment modality utilization between teaching and non-teaching hospitals reflects differences in resource availability and expertise. Teaching hospitals, often better equipped with advanced neurointerventional capabilities, perform more EVT procedures, while non-teaching and rural hospitals rely predominantly on IV-tPA due to limitations in infrastructure and specialist availability. There were no significant differences in utilization of these modalities based on primary insurance of the patients. EVT was more commonly utilized in low-income groups (Quartile 1 and Quartile 2), while IV-tPA was more frequently employed in high-income groups (Quartile 3 and Quartile 4); these differences in their utilization were statistically significant (Table 2).

**Table 2:** Baseline characteristics of VBAO hospitalizations.

Variables	No procedure^#^	IV-tPA	EVT	*p*-value
Age in years (%)				
18–44	87.8	5.2	7.0	0.0003
45–64	86.6	6.3	7.1	<0.0001
65–84	87.1	7.0	5.9	0.0008
> = 85	86.9	8.5	4.7	<0.0001
Gender (%)				
Male	87.2	6.6	6.2	0.18
Female	86.6	7.1	6.3	
Race (%)				
White	86.5	7.1	6.4	0.21
Black	88.3	5.4	6.3	0.0001
Hispanic	90.0	5.5	4.5	0.16
Others	83.5	9.5	7.0	0.02
Hospital bed size (%)				
Small	94.2	3.8	2.0	<0.0001
Medium	88.3	7.7	4.0	<0.0001
Large	84.9	7.0	8.1	<0.0001
Hospital region (%)				
Northeast	88.0	5.9	6.1	0.05
Midwest	87.7	5.9	6.4	0.002
South	87.8	6.3	5.9	0.3
West	83.6	9.5	6.9	<0.0001
Hospital type (%)				
Rural	96.7	2.7	0.7	<0.0001
Urban non-teaching	90.4	6.3	3.3	<0.0001
Teaching	67.8	5.6	5.7	<0.0001
Primary insurance (%)				
Medicare/Medicaid	87.3	6.6	6.2	0.09
Private including HMO	86.1	7.4	6.5	0.13
Uninsured/Self-pay	87.2	6.8	6.0	0.31
Median household income (%)				
Quartile 1	88.1	5.5	6.4	<0.0001
Quartile 2	88.5	5.5	6.0	0.0008
Quartile 3	86.4	7.5	6.1	0.007
Quartile 4	84.3	9.3	6.5	<0.0001
Disposition status (%)				
Home	90.8	6.3	2.9	<0.0001
Facility	86.8	6.8	6.4	0.23
Died	75.1	8.1	16.8	<0.0001
**Mean LOS (days ± SE)**	6.5 ± 0.1	7.3 ± 0.6	9 ± 0.5	0.62

**#“No procedure” column is for explanatory purposes only. Chi-square test was performed between IV t-PA and EVT groups.**

### Primary endpoints

Complications related to IV-tPA were cerebral edema (10.7%), ICH (2.4%), angioedema (0.4%), and hemoperitoneum (0.2%) (Table 3). Complications related to EVT were cerebral edema (19%), arterial dissection (8.5%), aneurysm of upper or lower extremities (0.7%), postprocedural hemorrhage or hematoma of skin and subcutaneous tissue following the procedure (0.6%), and postprocedural hemorrhage of a nervous system organ or structure following the procedure (0.4%) (Table 3).

**Table 3:** Complications: IV-tPA vs. EVT.

IV-tPA %		EVT %	
Cerebral edema	10.7	Cerebral edema	19
Intracerebral Hemorrhage	2.4	Arterial dissection	8.5
Angioedema	0.4	Aneurysm of artery of upper/lower extremity	0.7
Hemoperitoneum	0.2	Postprocedural hemorrhage/ hematoma of skin and subcutaneous tissue following a procedure	0.6
		Postprocedural hemorrhage of a nervous system organ or structure following a procedure	0.4

### Secondary endpoints

Compared to EVT, patients who received IV-tPA were more likely to be discharged to home (6.3% vs. 2.9%; *p* < 0.0001) (Table 2). The all-cause mortality rate was significantly higher in the EVT group compared to the IV-tPA group (16.8% vs. 8.1%, p < 0.0001). The mean LOS did not show a statistically significant difference between the two treatment modalities (Table 2).

### Factors associated with mortality

Multivariate logistic regression analysis identified significant factors associated with mortality in patients with VBAO. Patients aged 45–64 years (OR: 3.0, 95% CI: 1.4–6.4, *p* = 0.01) and 65–84 years (OR: 2.8, 95% CI: 1.3–5.9, p = 0.01) had higher odds of mortality compared to the reference group (aged 18–44 years). Gender did not significantly affect mortality risk, with females showing an OR of 1.2 (95% CI: 0.7–2.1, *p* = 0.56) compared to males. Race also did not show significant differences in mortality risk, with Hispanics (OR: 2.1, 95% CI: 0.4–10.9, *p* = 0.36), “Others” (OR: 1.3, 95% CI: 0.4–4.0, *p* = 0.68), and White patients (OR: 0.9, 95% CI: 0.4–2.0, *p* = 0.79) having no statistically significant differences compared to Black patients.

Hospital characteristics were another key factor, with rural hospitals showing a trend toward lower mortality (OR: 0.4, 95% CI: 0.0–3.0, *p* = 0.37) and urban non-teaching hospitals showing higher odds of mortality (OR: 1.3, 95% CI: 0.7–2.4, *p* = 0.34) compared to teaching hospitals, though these findings were not statistically significant. Notably, the presence of complications significantly reduced mortality risk (OR: 0.3, 95% CI: 0.2–0.6, *p* = 0.001). These findings, summarized in Table 4, highlight the complexity of mortality risk in VBAO patients and suggest that patient demographics, hospital characteristics, and clinical complications play a role in outcomes.

**Table 4:** Predictors of mortality.

Variables	OR	Confidence interval	*P*-value
Age			
18–44	2.3	0.7–7.5	0.18
45–64	3.0	1.4–6.4	0.01
65–84	2.8	1.3–5.9	0.01
> = 85	Control		
Gender			
Female	1.2	0.7–2.1	0.56
Male	Control		
Race			
White	0.9	0.4–2.0	0.79
Hispanic	2.1	0.4–10.9	0.36
Others	1.3	0.4–4.0	0.68
Black	Control		
Hospital type			
Rural	0.4	0.0–3.0	0.37
Urban non-teaching	1.3	0.7–2.4	0.34
Teaching	Control		
Any complication			
Yes	0.3	0.2–0.6	0.001
No	Control		

## Discussion

The efficacy of EVT for VBAO remains uncertain. Although VBAO is a rare form of stroke, comprising roughly 1% of all ischemic strokes and 5–10% of all proximal intracranial occlusions, its consequences are devastating. Without prompt treatment, around 70% of patients suffer from severe disability or face mortality (5). This study aimed to analyze the trends in hospitalizations for two treatment interventions, EVT and IV-tPA, for VBAO. The study also investigated the outcomes, complications, and factors associated with mortality.

The research involved a total of 37,310 patients who were admitted with VBAO between the years 2016 and 2018. Within this group, IV-tPA was administered in 6.8% of cases, while EVT was performed in 6.2% of cases. Comparatively, a previous study conducted in 2015 on the NIS cohort, which identified 1,120 patients with a diagnosis of basilar artery occlusion, reported an EVT rate of 16.43%. Despite our study encompassing both vertebral artery and basilar artery occlusions and utilizing more recent data from 2016 to 2018, our EVT utilization rate was lower. This disparity is challenging to explain, but it may be attributed to the inclusion of the subgroup with vertebral artery occlusion in our study (19).

The usage of IV-tPA and EVT demonstrated notable variations among different age groups. IV-tPA administration was more prevalent among patients aged 65 and above, whereas EVT was favored for individuals between 18 and 64 years old. The lower utilization of EVT in older age groups can be attributed to several factors. These include the higher occurrence of comorbidities that heighten the risk of complications, the presence of delicate blood vessels and intricate anatomy, as well as considerations regarding the overall health and functional status of elderly patients. It is important to acknowledge that clinical trials focusing on EVT in cases of VBAO, such as the ATTENTION, BEST, BASICS, and BAOCHE trials, have predominantly enrolled participants in their 60s, potentially excluding a significant portion of the elderly population (4, 5, 20, 21). Consequently, IV-tPA is frequently employed in this subgroup of patients.

Irrespective of hospital size, IV-tPA was more frequently employed than EVT for treating VBAO. In non-teaching hospitals, IV-tPA was the preferred treatment option, surpassing EVT in usage. However, in teaching hospitals, EVT was utilized more frequently than IV-tPA. These results highlight the complexity of treating VBAO and the critical influence of resource availability and patient factors on treatment decisions. For example, the preference for EVT in younger patients and at teaching hospitals suggests that procedural expertise and hospital infrastructure are significant determinants of treatment modality. Conversely, the reliance on IV-tPA in older populations and non-teaching hospitals underscores the challenges of accessibility to EVT in resource-limited settings. This emphasizes the need for a more equitable distribution of advanced neurointerventional services to ensure optimal stroke care across all demographics. This observation is consistent with a previous study conducted by Farooqui et al., which analyzed the NIS database and revealed that rural non-teaching hospitals did not perform any EVT procedures. The majority of EVT procedures were carried out in teaching hospitals (19). These findings support the notion that nonacademic hospitals lack the necessary capabilities for performing EVT and providing multidisciplinary critical care services. Consequently, these hospitals mainly handle mild to moderate stroke cases and refer severe cases to more specialized facilities with better resources.

In terms of outcomes, the study findings indicated that the all-cause mortality rate was notably higher in the EVT group compared to the IV-tPA group, with rates of 16.8 and 8.1%, respectively. It is important to note that the reported mortality rate in our study is lower than those reported in clinical trials (4, 5, 20, 21). Meta-analyses of these trials have demonstrated that EVT has a mortality rate of 35% compared to 45% for the best medical management option (22, 23). Furthermore, they have revealed that EVT is associated with a significantly reduced risk of mortality within 90 days in patients with VBAO when compared to the best medical management option (22, 23).

Our study did not find any notable disparity in the average duration of hospitalization between the two treatment approaches. However, a *post hoc* analysis of DEFUSE 3 revealed that the median length of hospital stay was significantly shorter in the EVT group, with patients in this group staying for an average of 6.5 days compared to 9.1 days in the medical group. Additionally, within the same study, EVT demonstrated an increase in the amount of time spent at home and improved living situations for patients during the 90-day period following a stroke (24). Another study demonstrated that individuals who underwent thrombectomy were discharged, on average, 36 days earlier. Moreover, a greater number of patients were able to return home upon discharge, while fewer individuals required placement in care homes or rehabilitation centers. Furthermore, the thrombectomy group necessitated a reduced number of physiotherapy sessions (25).

The research also investigated the potential complications associated with EVT for the treatment of VBAO. The identified complications included cerebral edema, arterial dissection, aneurysm formation in the upper or lower extremities, as well as postprocedural hemorrhage or hematoma. The complications associated with EVT were discussed broadly in this study; however, it is important to recognize that not all complications contribute equally to morbidity or mortality. While complications such as vessel perforation or arterial dissection can result in catastrophic outcomes, others like pseudoaneurysms or minor vascular access complications are typically of lower clinical significance and rarely lead to mortality. The higher mortality rate observed in EVT compared to IV-tPA is likely multifactorial, involving both procedural complications and baseline differences in patient severity that were not fully accounted for due to the limitations of the dataset. This distinction underscores the importance of separating major complications, which directly impact survival, from minor complications that primarily affect morbidity. While EVT is generally considered a safe technique, there is a potential risk of bleeding. Manipulation of the vessel wall during the procedure, especially using devices, can result in vessel damage and subsequent intimal injury. This damage can range from subintimal dissection to the formation of an intramural hematoma, which may eventually lead to the occlusion of the treated vessel. The extent of the damage may vary based on factors such as the design of the device, the number of device passes, and the diameter of the target vessel.

The differences observed between our study findings and those from previous clinical trials, such as the BASICS, BEST, ATTENTION, and BAOCHE trials, can be attributed to several factors. First, our study utilized a real-world, population-based dataset from the NIS, which includes a broader and more diverse patient population compared to the highly selective cohorts enrolled in clinical trials. For instance, clinical trials often exclude elderly patients or those with significant comorbidities, whereas the NIS database captures a wide range of patient demographics and clinical presentations. This inclusivity may explain the lower overall rates of EVT observed in our study compared to trials like BEST and BASICS, which focused on carefully selected patients with favorable characteristics.

Second, differences in study design contribute to the observed discrepancies. Clinical trials are conducted in controlled environments with standardized protocols and expert centers, ensuring uniformity in treatment practices. In contrast, the NIS database reflects real-world variability in resource availability, procedural expertise, and adherence to guidelines across different hospital types and regions. For example, our finding that EVT utilization is higher in teaching hospitals highlights the influence of institutional capabilities on treatment practices, a factor not typically accounted for in clinical trials.

Third, the absence of granular clinical data in the NIS database, such as imaging findings, stroke severity scores (e.g., NIHSS), and precise treatment timing, limits direct comparisons with clinical trials, which rely on such detailed metrics to define eligibility and assess outcomes. The absence of this data may lead to residual confounding and limits the ability to fully align our results with those of clinical trials.

### Strengths of the study

- The utilization of the NIS database enabled us to conduct a study on a substantial population, thereby mitigating the bias commonly observed in studies limited to a single region or hospital.
- Our analysis provides a framework for future clinical trials to compare outcomes for IV-tPA versus EVT.

### Limitations of the study

- Due to the limited scope of NIS, our assessment of 90-day outcomes was not possible as NIS solely focuses on inpatient populations.
- We could not assess rates of complications related to EVT like distal embolization, vessel rupture, vessel re-occlusion post mechanical thrombectomy, post reperfusion injury, seizures. Also, we could not calculate rates of access site related complications like vessel occlusion, arterio-venous fistula, rectus sheath hematoma, femoral neuropathies, and infections, and treatment outcomes like hemorrhagic infarction, intraparenchymal hemorrhage, and subarachnoid hemorrhage.
- The number of patients with VBAO in our analysis may be overestimated. This is because the NIS treats each hospitalization as a distinct entry, without a coding method to differentiate between initial admissions and subsequent readmissions.
- Major limitation of this study is the inability to account for stroke severity directly, as the NIS database does not include measures such as the NIHSS. Stroke severity is a critical mediator in the selection of IV-tPA versus EVT and significantly impacts outcomes. Although we used proxy variables such as age, the presence of complications (e.g., cerebral edema and hemorrhage), and hospital characteristics to partially account for severity, these proxies are not a substitute for direct clinical measures. This limitation may result in residual confounding and should be considered when interpreting the study’s findings.
- Another important limitation of our study is the absence of data on the time interval between the diagnosis of VBAO and the initiation of EVT or IV-tPA treatment. The timing of treatment initiation is a critical determinant of outcomes in acute ischemic stroke, as earlier intervention within the therapeutic window is associated with better functional outcomes and reduced mortality. Unfortunately, the NIS database does not capture this information, which limits our ability to evaluate the time-dependent efficacy of these treatment modalities. Future studies leveraging datasets with detailed timing variables are necessary to provide a more comprehensive understanding of the real-world effectiveness of EVT and IV-tPA.

### Future directions

To further explore the treatment of VBAO, future research should focus on the following:

- Understanding clot composition and its effects on treatment efficacy. This entails analyzing clot characteristics like size and density, and their responsiveness to treatments like EVT and IV-tPA.
- Assessing diverse patient demographics to understand how factors like age and comorbidities influence treatment outcomes.
- Integrating advanced imaging techniques to enhance diagnostic precision and guide treatment choices.
- Exploring new therapies alongside EVT and IV-tPA, and investigating the potential of AI and machine learning in personalizing treatment and improving diagnostic accuracy.
- Understanding the underlying factors influencing treatment decisions, such as hospital infrastructure, socioeconomic disparities, and referral pathways, to identify strategies for improving access to EVT for underserved populations.
- Incorporating clinical measures, such as NIHSS scores, stroke risk factors, baseline functional status, and stroke mechanisms, to provide a more comprehensive understanding of the comparative safety and efficacy of IV-tPA and EVT.
- Utilizing longitudinal datasets or prospective designs that include 90-day outcomes, such as functional status and recurrent events, to provide a more complete understanding of the long-term efficacy and safety of IV-tPA and EVT.

## Conclusion

This study highlights significant trends and outcomes associated with EVT and IV-tPA in patients with VBAO, shedding light on the variability in treatment utilization and associated mortality rates. However, the findings should be interpreted with caution, considering the inherent limitations of the NIS database, such as the absence of critical time-dependent and clinical severity variables, as well as the lack of long-term outcome data. While these results provide valuable real-world insights, they are restricted in scope and should not be generalized without validation from prospective studies or clinical trials. Further research is warranted to address these gaps, explore the long-term efficacy and safety of these treatment modalities, and assess their impact in diverse patient populations and healthcare settings.

## Data Availability

The data analyzed in this study is subject to the following licenses/restrictions: In order to reproduce the data used in our research, you have get access from HCUP directly. Requests to access these datasets should be directed to https://hcup-us.ahrq.gov/team/NationwideDUA.jsp.
