# Supplementary material for: Endovascular thrombectomy versus intravenous tissue plasminogen activator for vertebrobasilar stroke treatment: insights from the national inpatient sample
Source: Front Neurol. 2025 Apr 24;16:1417188. doi: 10.3389/fneur.2025.1417188 (PMC12071085; doi:10.3389/fneur.2025.1417188)
Supplement: Supplementary file 1 [file Table_1.docx]

**ICD-10-CM codes for diagnosis and procedures**

| **Diagnosis** | **ICD-10-CM diagnoses code** | **Description of code** |
| --- | --- | --- |
| VBAO | I63.21 | Cerebral infarction due to unspecified occlusion or stenosis of vertebral arteries |
|  | I63.22 | Cerebral infarction due to unspecified occlusion or stenosis of basilar artery |
|  | I63.01 | Cerebral infarction due to thrombosis of vertebral artery |
|  | I63.02 | Cerebral infarction due to thrombosis of basilar artery |
|  |  |  |
| **Procedures** | **ICD-10-CM procedure code** | **Description of code** |
| IV tPA | 3E03317 | Introduction of Other Thrombolytic into Peripheral Vein, Percutaneous Approach |
| EVT | 03CG3ZZ | Extirpation of Matter from Intracranial Artery, Percutaneous Approach |
|  | 03CG3Z7 | Extirpation of Matter from Intracranial Artery using Stent Retriever, Percutaneous Approach |
|  | 03CG4ZZ | Extirpation of Matter from Intracranial Artery, Percutaneous *Endoscopic* Approach |
|  | 03CG3Z6 | Extirpation of Matter from Intracranial Artery, Bifurcation, Percutaneous Approach |
|  | 03CG4Z6 | Extirpation of Matter from Intracranial Artery, Bifurcation, Percutaneous *Endoscopic* Approach |

**ICD-10-CM codes for complications**

| **IV-tPA** | | **EVT** | |
| --- | --- | --- | --- |
| **Complication** | **ICD-10-CM-codes** | **Complication** | **ICD-10-CM codes** |
| **Non-traumatic cerebral edema** | G93.6 | **Non-traumatic cerebral edema** | G93.6 |
| **Intracerebral Hemorrhage** | I61.9 | **Arterial dissection** | I77.75 - Dissection of other precerebral arteries  I77.74 - Dissection of vertebral artery  I77.71 - Dissection of carotid artery |
| **Angioedema** | T78.3 | **Aneurysm of artery of upper/lower extremity** | I72.1  I72.4 |
| **Hemoperitoneum** | K66.1 | **Postprocedural hemorrhage/hematoma of skin and subcutaneous tissue following other procedure** | L76.22  L76.32 |
|  |  | **Postprocedural hemorrhage of a nervous system organ or structure following a nervous system procedure** | G97.51 |
