# Supplementary material for: Endovascular thrombectomy versus intravenous tissue plasminogen activator for vertebrobasilar stroke treatment: insights from the national inpatient sample
Source: Front Neurol. 2025 Apr 24;16:1417188. doi: 10.3389/fneur.2025.1417188 (PMC12071085; doi:10.3389/fneur.2025.1417188)
Supplement: Supplementary file 2 [file Table_2.docx]

**DESCRIPTION OF VARIABLES**

1. **Bed size**

| **Location and Teaching Status** | **Hospital bed size** | | |
| --- | --- | --- | --- |
|  | **Small** | **Medium** | **Large** |
| **NORTHEAST REGION** | | | |
| Rural | 1-49 | 50-99 | 100+ |
| Urban, nonteaching | 1-124 | 125-199 | 200+ |
| Urban, teaching | 1-249 | 250-424 | 425+ |
| **MIDWEST REGION** | | | |
| Rural | 1-29 | 30-49 | 50+ |
| Urban, nonteaching | 1-74 | 75-174 | 175+ |
| Urban, teaching | 1-249 | 250-374 | 375+ |
| **SOUTHERN REGION** | | | |
| Rural | 1-39 | 40-74 | 75+ |
| Urban, nonteaching | 1-99 | 100-199 | 200+ |
| Urban, teaching | 1-249 | 250-449 | 450+ |
| **WESTERN REGION** | | | |
| Rural | 1-24 | 25-44 | 45+ |
| Urban, nonteaching | 1-99 | 100-174 | 175+ |
| Urban, teaching | 1-199 | 200-324 | 325+ |

1. **Region**

| **Region** | **States** |
| --- | --- |
| Northeast | ME, NH, VT, MA, RI, CT, NY, NJ, and PA |
| Midwest | OH, IN, IL, MI, WI, MN, IA, MO, ND, SD, NE, and KS |
| South | DE, MD, DC, VA, WV, NC, SC, GA, FL, KY, TN, AL, MS, AR, LA, OK, and TX |
| West | MT, ID, WY, CO, NM, AZ, UT, NV, WA, OR, CA, AK, and HI |

1. **Insurance status**

| **Primary payer** | **Description** |
| --- | --- |
| Medicare | Fee-for-service and managed care Medicare patients |
| Medicaid | Fee-for-service and managed care Medicaid patients |
| Private insurance | Blue cross, Blue shield, HMO (Health Maintenance Organization) plans, PPO (Preferred Provider Organization) plans, and other private insurance providers |
| Uninsured | Self-pay/No-charge |

1. **Median household income**

| **Quartile** | **Description** |
| --- | --- |
| 1 | 0-25th percentile |
| 2 | 26th to 50th percentile (median) |
| 3 | 51st to 75th percentile |
| 4 | 76th to 100th percentile |
